# Spatial-Temporal Data Collection with Compressive Sensing in Mobile Sensor Networks

**DOI:** 10.3390/s17112575

**Published:** 2017-11-08

**Authors:** Haifeng Zheng, Jiayin Li, Xinxin Feng, Wenzhong Guo, Zhonghui Chen, Neal Xiong

**Affiliations:** 1 College of Physics and Information Engineering, Fuzhou University, Fuzhou 350116, China; zhenghf@fzu.edu.cn (H.Z.); N151120061@fzu.edu.cn (J.L.); fxx1116@fzu.edu.cn (X.F.); czh@fzu.edu.cn (Z.C.); 2 Fujian Provincial Key Laboratory of Network Computing and Intelligent Information Processing, Fuzhou University, Fuzhou 350116, China; 3 Key Laboratory of Spatial Data Mining & Information Sharing, Ministry of Education, Fuzhou 350116, China; 4 Department of Mathematics and Computer Science, Northeastern State University, Muskogee, OK 74401, USA

**Keywords:** compressive sensing, mobile data gathering, machine learning theory, random walk, Gaussian kernel, wireless sensor networks

## Abstract

Compressive sensing (CS) provides an energy-efficient paradigm for data gathering in wireless sensor networks (WSNs). However, the existing work on spatial-temporal data gathering using compressive sensing only considers either multi-hop relaying based or multiple random walks based approaches. In this paper, we exploit the mobility pattern for spatial-temporal data collection and propose a novel mobile data gathering scheme by employing the Metropolis-Hastings algorithm with delayed acceptance, an improved random walk algorithm for a mobile collector to collect data from a sensing field. The proposed scheme exploits Kronecker compressive sensing (KCS) for spatial-temporal correlation of sensory data by allowing the mobile collector to gather temporal compressive measurements from a small subset of randomly selected nodes along a random routing path. More importantly, from the theoretical perspective we prove that the equivalent sensing matrix constructed from the proposed scheme for spatial-temporal compressible signal can satisfy the property of KCS models. The simulation results demonstrate that the proposed scheme can not only significantly reduce communication cost but also improve recovery accuracy for mobile data gathering compared to the other existing schemes. In particular, we also show that the proposed scheme is robust in unreliable wireless environment under various packet losses. All this indicates that the proposed scheme can be an efficient alternative for data gathering application in WSNs.

## 1. Introduction

Wireless sensor networks have been widely deployed in a variety of applications including environmental monitoring, traffic surveillance and social sensing and analysis [1,2,3,4]. In such networks, data gathering is one of most fundamental tasks, where a large amount of sensory data is required to be transmitted to a fusion center (FC). However, owing to power limitation and resource constraints of sensor nodes, it is impractical for them to directly transfer all of data to the FC without any dimensionality reduction. Since sensory data collected in many scenarios intrinsically exhibits spatial-temporal correlations, some conventional data gathering schemes have been proposed to reduce energy consumption of data transmissions [5,6,7,8]. Recently, compressive sensing provides a new approach for data gathering applications in WSNs, which allows for the original signal recovery from a small number of measurements as long as the signal can be sparsely represented in a certain transform domain [9]. Compressive sensing is able to perform sensing and compression simultaneously to reduce the amount of data transmitted over the network so as to save energy consumption at each sensor node.

Various compressive sensing based approaches have been investigated for efficient data gathering in WSNs [10,11,12,13,14,15,16,17,18,19,20,21,22,23,24,25,26,27]. However, most of the current CS-based data gathering schemes try to exploit either spatial or temporal correlation among nodes. Thus, the performance improvement brought by CS is limited. Considering many sensor network applications in practice especially in environmental monitoring, sensory data are usually periodically collected for a long time. Thus, the temporal correlation at each sensor node can be further exploited. Consequently, one can take full advantage of both spatial and temporal correlations to further improve the data gathering performance.

Recently, some CS-based data gathering schemes have been proposed to exploit both spatial and temporal correlations of sensory data in WSNs [28,29,30,31,32]. (See Section 2 for more discussions.) For example, Mahmudimanesh et al. proposed a balanced temporal-spatial CS scheme by applying a joint sparsity model to improve the reconstruction accuracy of the spatial-temporal profile of WSNs [28]. Quer et al. proposed an effective framework for data gathering to exploit spatial and temporal correlations by jointly using CS and Principle Component Analysis (PCA) [29]. These two schemes above mainly utilize the spatial-temporal correlation characteristic of the signal in the recovery algorithm to improve the reconstruction accuracy. Xu et al. proposed a spatial-temporal hierarchical data aggregation scheme using compressive sensing, where a subset of nodes is randomly selected to collect the corresponding subset data at each data collection instant and then matrix completion algorithm is applied to reconstruct all the data of the entire network [30]. In this scheme, some specific nodes or cluster heads are designated to forward data to the FC, which inevitably leads to unbalanced energy consumption. Wang et al. proposed a data gathering scheme based on Kronecker compressive sensing theory by exploiting both temporal and spatial sparsity of sensory data to improve reconstruction accuracy [31]. In this scheme, for each data gathering period, only a subset of nodes is randomly selected to transmit their CS measurements to the FC.

Mobile data gathering has also attracted much attention in the past few years, where one or more mobile collectors are designated to take responsibility for collecting data from sensor nodes [33,34,35,36,37,38]. The reason to use mobile collector(s) for data gathering is to reduce energy expenditure at each node. Since a mobile collector can come closely to a node for data collection, it is not necessary for a node to use a powerful transceiver to communicate with the mobile collector. Instead, a node only needs to wait for the mobile collector to get close enough, which can significantly reduce power for communication. Furthermore, it is also not necessary for all of nodes to maintain the connectivity of an entire network since they only need to communicate with the mobile collector(s) instead of with the other nodes. Due to the merits of mobile data gathering, the mobility pattern combining with compressive sensing has been exploited for data gathering in several works [24,25,26,27,39,40,41]. However, previous work above does not exploit spatial-temporal correlation of sensory data.

In this paper, we propose a novel mobile data gathering scheme by exploiting spatial-temporal correlation of sensory data to improve the performance of energy consumption and signal recovery quality in WSNs. The contributions of this paper are summarized as follows.

- We propose a data gathering scheme using the Metropolis-Hastings algorithm with delayed acceptance (MHDA), a random walk algorithm for a mobile collector to collect data from sensor nodes. In this scheme, the mobile collector only needs to collect temporal CS measurements from a subset of nodes by sequentially visiting these nodes along a random routing path. Unlike [26,27] which need to perform multiple random walks to obtain random projections, the mobile collector takes only one random walk to collect measurements from nodes, which can significantly reduce the cost of data transmissions and improve energy efficiency. On the other hand, instead of using a standard random walk as [41], we adopt an improved Metropolis-Hastings algorithm as a mobility pattern which results in more uniform sampling distribution to improve signal reconstruction accuracy.
- We prove that the sensing matrix for spatial dimensional signal which is constructed from the proposed random walk algorithm and a kernel-based sparsity representation basis satisfies the Restricted Isometry Property (RIP). We also exploit KCS for spatial-temporal correlation of sensory data to improve the compression performance and prove that the equivalent sensing matrix for spatial-temporal dimensional signal can satisfy the property of KCS models.
- We also show that the proposed scheme is robust to the packet loss environment. We present simulation results to demonstrate that the proposed scheme is able to not only reduce communication cost but also improve recovery accuracy for mobile data gathering compared to some existing schemes.

The remainder of this paper is organized as follows. In Section 2, we introduce the related work. In Section 3, the preliminaries on CS basics and system model are given and the problem is formulated. In Section 4, we present a random walk algorithm with CS for mobile data gathering and the design for measurement matrix and sparsity representation basis. In Section 5, we give theoretical analysis and the performance analysis for the proposed scheme. In Section 6, we conduct extensive simulations to evaluate the performance of the proposed scheme. Finally, we draw a conclusion and discuss our future work in Section 7.

## 2. Related Work

In the past few years, much research work has been done to investigate the efficiency of compressive sensing for data gathering in WSNs [10,11,12,13,14,15,16]. For example, Luo et al. proposed a CS-based data gathering scheme for large-scale sensor networks. The goal of this work is to improve network capacity by alleviating the bottleneck effect of the sink. On the other hand, there are much work dedicated to improve the energy efficiency of data gathering by employing CS technique [10]. For instance, Xiang et al. also proposed a data gathering scheme to improve energy efficiency, where the entire network is partitioned into subnetworks and CS is employed within each subnetwork for data gathering [12]. Zhao et al. proposed a treelet-based clustered data gathering scheme to save energy expenditure, where a treelet transform is adopted as a sparsity representation basis and then CS is applied in conjunction with a clustered routing algorithm [16]. Furthermore, another research line for improving energy efficiency of data gathering by using CS is to design sparse random measurement matrices [19,20,21,22]. For instance, Zheng et al. designed a new type of random measurement matrix by using random walk algorithm for data gathering which follows the theory of expander-based compressive sensing [21]. Liu et al. proposed a non-uniform sparse random matrix by constructed from an opportunistic routing algorithm [22]. Meanwhile, Nguyen et al. exploited an integration between CS and the random mobility of sensors in distributed mobile sensor networks [26,27,39]. In their work, a small number of mobile sensors are utilized to collect data at their random positions and exchange their readings with their neighbors within the sensor transmission range to form one CS measurement. Rana et al. proposed a method for the mobile nodes to adaptively predict the number of projections based on the speed of the mobile nodes and compute a deterministic projection matrix from a learnt dictionary [40]. However, the aforementioned work only considers spatial correlation of sensing field by using CS.

Recently, some work has been dedicated to exploiting both spatial and temporal correlations of sensory data to improve the performance of data gathering in WSNs. For example, Mahmudimanesh et al. proposed a balanced temporal-spatial CS scheme by applying a joint sparsity model to improve the reconstruction accuracy of the spatial-temporal profile of WSNs [28]. Quer et al. propose an effective framework for data gathering by exploiting spatial and temporal correlations jointly using CS and Principle Component Analysis (PCA), where they develop a sparse sampling matrix [29]. Different from their work, we study on how to collect temporal-spatial sensory data with a mobile collector whereas they focus on improving the recovery quality on the reconstruction side by exploiting spatial and temporal correlations using PCA. Xu et al. proposed a spatial-temporal hierarchical data aggregation scheme using compressive sensing, where a subset of nodes is randomly selected to collect the corresponding subset data at each data collection instant and then matrix completion algorithm is applied to reconstruct all the data of the entire network [30]. In their work, they adopted a multi-level clustering fashion to collect spatial-temporal data. Since random projections are forwarded to the FC along the tree, any packet loss at some specific nodes or cluster heads during transmission will results in the loss of previously calculated random projection. Thus, the value of random projection obtained at the FC is inaccuracy. Consequently, such an approach is susceptible to packet loss. Wang et al. proposed a data gathering strategy based on KCS theory by exploiting both temporal and spatial sparsity of sensory data to improve reconstruction accuracy [31]. Li et al. proposed a CS-based data gathering algorithm which utilize random sampling and random walks to select sensory data in temporal and spatial domains [32]. In their work, each projection is obtained by summing the received measurements and the sensing matrix is designed based on the adjacency matrix of an unbalanced expander graph. Different from the above work, we exploit the mobility pattern for spatial-temporal data collection, where it allows the mobile collector to gather data by sequentially visiting a subset of nodes, thus significantly reducing energy expenditure of sensor nodes. Furthermore, in our scheme a projection is generated from only one sensor node instead of a linear combination of the measurements from multiple nodes.

## 3. Preliminaries and Problem Formulation

### 3.1. Compressive Sensing Basics

Compressive sensing provides a new paradigm for signal sampling and compression. CS theory asserts that a sparse or compressible signal can be reconstructed with high probability from a small number of measurements, which is far smaller than the length of the original signal. For example, consider an *n*-dimensional signal vector $x={\left(x_{1},\ldots ,x_{n}\right)}^{T}$. We say that x is a *k*-sparse signal if there are at most k(k≪n) nonzero elements in x.

To reduce the dimensionality of x, CS applies a measurement matrix $\Phi \in R^{m\times n}$ onto x to obtain an *m*-dimensional signal $y\in R^{m}$. The measurement matrix Φ can be a Gaussian or Bernoulli random matrix which follows the restricted isometry property (RIP) [42]. CS theory states that the *k*-sparse signal x can be accurately recovered with high probability from m=O(klog(n/k)) linear combinations of measurements y. It has been proven that recovering the signal x from y can be achieved through solving an $\ell_{1}$-minimization problem [43]:

(1) $$\min_{x\in {ℜ}^{n}}{∥x∥}_{\ell_{1}}s.t.\;y=\Phi x.$$

However, the signals existing in nature, such as temperature and humidity, are not perfectly *k*-sparse as they may have *k* large transform coefficients while the remaining coefficients are small. Suppose that the signal x can be represented $\Psi =(\psi_{1},\ldots ,\psi_{n})$ as

(2) $$x=\Psi \theta =\sum_{i=1}^{n}\theta_{i}\psi_{i},$$

in an n×n orthogonal basis Ψ, where $\theta ={\left(\theta_{1},\ldots ,\theta_{n}\right)}^{T}$ is the transform coefficients of x in the basis Ψ.

We reorder the coefficients $\theta_{i}$ in decreasing magnitude such that

(3) $$|\theta_{1}\left|\geq \right|\theta_{2}\left|\geq \right|\theta_{3}\left|\geq \ldots \geq \right|\theta_{n}|.$$

We say that the signal x is a power-law decay signal in the basis Ψ if for some fixed *p*, the *i*th largest transform coefficient satisfies

(4) $$|\theta_{i}|\leq Ri^{-1/p},R>0,p\in \left(0,1\right]$$

for each 1≤i≤n, where *p* controls the compressibility of the transform coefficients (i.e., a smaller *p* implies faster decay) and *R* is a constant. The best *k*-term approximation of x (obtained by keeping the *k* largest coefficients and setting the others to zero) is given by ${\hat{x}}_{k}=\sum_{i=1}^{k}\theta_{i}\psi_{i}$, where 1≤k≤n is fixed. We say that x is compressible in Ψ when the mean squared approximation error behaves like

(5) $$∥x-{\hat{x}}_{k}{∥}_{2}^{2}\leq C_{r}R^{2}k^{-r},\;r=2/p-1,$$

for some fixed constant $C_{r}>0$ that only depends on *p*, where the parameter *p* controls the compressibility of x in Ψ. CS theory states that the compressible signals can also be optimally recovered from m=O(klog(n/k)) random measurements with high probability [42] through solving an $\ell_{1}$-minimization problem [43]:

(6) $$\min_{\theta \in {ℜ}^{n}}{∥\theta ∥}_{\ell_{1}}s.t.\;y=\Phi x,x=\Psi \theta .$$

### 3.2. System Model

We consider a multi-hop wireless sensor network for data gathering, which consists of *n* sensor nodes N=(1,…,n) and one mobile collector C. We assume that the sensor nodes are deployed uniformly and randomly in a unit square area to periodically sample spatial-temporal data from a sensing field. Suppose that the *i*th sensor node takes *m* readings for every sensing period *T* at a certain sampling speed in a data gathering tour, which is denoted as a signal vector $x_{i}=\left(x_{1,i},x_{2,i},\ldots ,x_{m,i}\right)$. In this paper, we model the WSN as a random geometric graph G(V,E), where V is a set of vertices representing the sensor nodes N and E is a set of edges representing the links among the sensor nodes.

**Figure 1:**
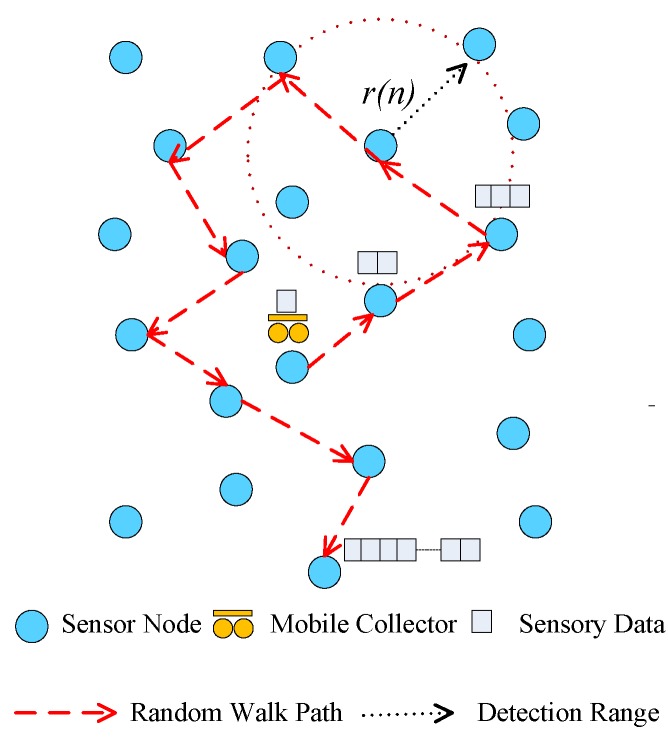
An example of data collection with a mobile collector in a WSN.

Consider a mobile collector roaming over the graph *G*. The mobile collector can be a physical mobile agent (e.g., data mule) [44]. We assume that the mobile collector can visit a sensor node to collect data at time *t* and then moves to one of its neighbors within its detection range r(n) at time t+1. The detection range is defined as the maximal distance that the mobile collector can move in each time slot. Figure 1 illustrates an example of collecting sensory data in a WSN. In this work, sensory data collected by the mobile collector at each node comes from the compressive temporal measurements of a node in a sensing period.

A similar model for mobile data gathering in WSNs was adopted in [37]. We assume that there is an edge between two sensor nodes if the Euclidean distance between them is smaller than the distance r(n). One of the merits to use the mobile collect is due to the fact that it does not need to equip with a powerful transceiver to communicate with sensor nodes. Instead, the mobile collect can roam closely to sensor nodes (much smaller than r(n)), which can also significantly reduce energy budget at each sensor node.

### 3.3. Problem Formulation

In this paper, we assume that the sensory data exhibits both spatial and temporal correlations, which is typical for most environmental monitoring applications in a densely deployed WSN. Let $D\in \;R^{m\times n}$ represent spatial-temporal sensory data collected by *n* sensor nodes in every sensing period *T*, where the *i*th column of *D* is data vector sampled by the *i*th sensor node during the sensing period *T* and the *j*th row of *D* is data vector sensing by *n* sensor node at a sampling instant $T_{j}$. We also assume that the signal *D* is sparse in both spatial domain $\Psi_{s}$ and temporal domain $\Psi_{t}$. It has been proven that by using a certain type of sparse projection matrix the information of the entire sensing field can be approximately reconstructed from only a small fraction of randomly selected sensor nodes from the perspective of CS theory [23]. As discussed above, if these random selected sensor nodes directly transmit their data to the FC through multi-hop, it will consume a large amount of energy budget. Therefore, in this paper we consider a random walk based strategy for the mobile collector to travel over the graph *G* to collect spatial-temporal data from a small subset of sensor nodes. We tackle the following problems: (1) What is the appropriate movement strategy (i.e., the transition probability of a random walk ) for the mobile collector so that the sensing field can be reconstructed from a small fraction of sensor nodes? (2) How many nodes and how many measurements at each node should be collected in a data gathering period? (3) How many steps for the mobile collector should take to collect these measurements in a data gathering period?

## 4. Design of a Mobile Data Gathering Scheme with Compressive Sensing

### 4.1. Spatial-Temporal Data Gathering with Random Walk

In this section, we propose a random walk based scheme for spatial-temporal data gathering in a WSN. In every period *T* of data gathering, after sampling *m* measurements, each sensor node applies CS to compress temporal sensory data using a random projection matrix $\Psi_{t}$, i.e., $y_{i}=\Psi_{t}x_{i}$, where $y_{i}$ is the random projections of the *i*th sensor node and $\Psi_{t}\in R^{m_{t}\times m}$ can be a Gaussian or Bernoulli random matrix. We now describe how to employ mobile collector with a random walk algorithm to gather random projections from a fraction of sensor nodes. We adopt an improved Metropolis-Hastings random walk algorithm (MHRW), named Metropolis-Hastings random walk algorithm with delayed acceptance (MHDA) [45]. The MHDA algorithm not only achieves a uniform stationary distribution for the mobile collector to unbiasedly visit sensor nodes but also improves efficiency comparing with the traditional MH algorithm. Specially, under the MHDA algorithm, the mobile collector reduces the bias of visiting previous nodes when choosing its next step. Different from MHRW, MHDA avoids the random walk to backtrack to the previous visited node. The idea using the MHDA algorithm for mobile data gathering is described as follows. At the beginning of the algorithm, the mobile collector C randomly selects one of sensor node i∈N to invoke a random walk with the length t=0 and collect random projections $y_{i}$ from node *i*. At time *t*, the mobile collector C chooses a node *j* uniformly at random from its neighbors of the current visited node *u* within its detection range r(n) to visit. MHDA considers two scenarios for the mobile collector to harvest data when it selects the next step. Let $Y_{t}$ and *k* be the previous visited node and the next node to be visited, respectively. First, if *j* is the previously visited node $Y_{t}$, then the walk will stop going to *j* and the transition to node *j* is delayed. Instead, it will go to another node k∈N(u)\{j} with another proposal probability 1/(d(u)−1), where N(u) is the set of the neighbor nodes of *u* and d(u) denotes the degree of node *u*. The proposed transition to *k* is then accepted with another acceptance probability [45]

(7) $$A^{\prime }\left(u,k\right)=\min\left\{1,\min\left\{1,{\left(\frac{d(u)}{d(k)}\right)}^{2}\right\}\max\{1,{\left(\frac{d(j)}{d(u)}\right)}^{2}\right\}\}.$$

**Figure 2:**
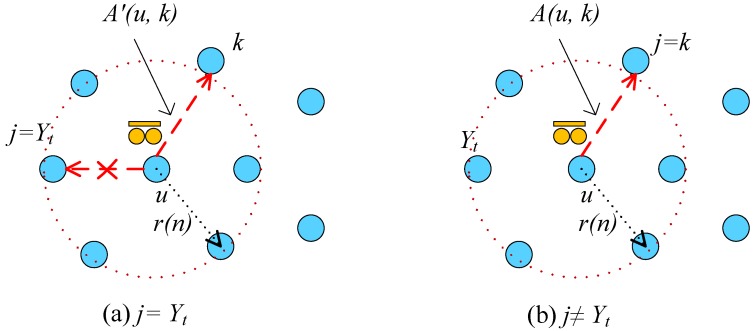
Illustrating two scenarios for MHDA algorithm. (**a**) The next node *j* is the previous visited node $Y_{t}$; (**b**) The next node *j* is not the previous visited node $Y_{t}$.

It has been proved in [45] that when the acceptance probability $A^{\prime }\left(u,k\right)$ is chosen as the condition in Equation (7) then the transition matrix P=[P(i,j)] of the random walk is irreducible and non-reversible with a unique stationary distribution and leads to the unbiased estimator. Second, if $k\neq Y_{t}$, then the walk will go to *k* to collect data as in the traditional MHRW algorithm. The proposed state transition to *k* is accepted with an acceptance probability $A\left(u,k\right)=\min\left\{1,\frac{d(u)}{d(k)}\right\}$. Figure 2 shows two scenarios for MHDA algorithm. At each time, when the walk visits a new node, the node sends its random projections to the mobile collector and increments the length *t*. When *t* reaches a given quantity, then the mobile collector C stops the algorithm and performs the CS recovery algorithm. The procedure of the mobile collector employing the MHDA algorithm is summarized in Algorithm 1.

In the proposed scheme, it requires accurate location information of sensor nodes to help the mobile collector to find the nodes to be visited at each step. On the other hand, these location information is also needed to construct Gaussian kernel basis required for the recovery algorithm. Generally, there are mainly two categories to obtain location information in WSNs: range-free and range-based approaches. Range-free approaches provide imprecise estimation of the node location, which mainly rely on connectivity measurements (e.g., hop count), whereas range-based approaches provide more precise location estimation by measuring the Euclidean distances among the nodes with various ranging techniques such as TOA (time of arrival), TDOA (difference of arrival), AOA (angle of arrival) and RSSI (received signal strength indicators). One also can combine range-free and range-based approaches to improve the localization accuracy [46]. On the other hand, for an outdoor environment, the mobile collector can be equipped with a GPS module to obtain the location information. With the location information, the mobile collector can get closely to a node for data collection. For the recovery algorithm of CS, the mobile collector should know which nodes have been visited. Thus, the additional overhead for the mobile collector only needs to contain the corresponding indices of the nodes to indicate which nodes have been visited. We also note that this overhead does not increase with the size of sensory data collected by the mobile collectors and only depends on the number of sensor nodes in the network. For example, in practice, we only need one bit for each sensor. Therefore, the additional overhead for the random walk is very small compared to the size of data sensory collected by the mobile collector, which can be neglected.

**Algorithm 1** The MHDA random walk algorithm for mobile data gathering at time *t*.1:Select a node *j* uniformly at random from neighbors of *u*, i.e, N(u)2:Generate a uniform random probability p∈[0,1]3:**if** p<min{1,d(u)/d(j)} **then**4: **if** node *j* is the previous node $Y_{t}$ (i.e., $Y_{t}=j$) and d(u)>1 **then**5:  select a node *k* uniformly at random from neighbors N(u)\{j}6:  Generate a uniform random probability q∈[0,1]7:  **if** $q<\min\{1,\min\left\{1,{\left(d\left(u\right)/d\left(k\right)\right)}^{2}\right\}\max\{1,{\left(d\left(j\right)/d\left(u\right)\right)}^{2}\}\}$ **then**8:   C visits node *k* and collects random projections $y_{k}$ from node *k*9:   $B_{t+1}=B_{t}\cup y_{k}$ and $Y_{t+1}\leftarrow i$10:  **else**11:   C visits node *j* and collects random projections $y_{j}$ from node *j*12:   $B_{t+1}=B_{t}\cup y_{j}$ and $Y_{t+1}\leftarrow j$13:  **end if**14: **else**15:  C visits node *j* and collects random projections $y_{j}$ from node *j*16:  $B_{t+1}=B_{t}\cup y_{j}$ and $Y_{t+1}\leftarrow j$17: **end if**18:**else**19: Stay at node *u* and $Y_{t+1}\leftarrow Y_{t}$20:**end if**

### 4.2. Random Measurement Matrices Design

In this section, we study the random matrices used in the proposed scheme. As discussed above, the random matrix $\Phi_{t}$ for temporal data can be a Gaussian or Bernoulli random matrix. In the following, we study the random matrix constructed from the MHDA algorithm. Let $\Phi_{s}$ be an $m_{s}\times n$ boolean random matrix. Each row of $\Phi_{s}$ contains only one nonzero element, i.e., “1”, which denotes that the corresponding sensor node has been visited by the mobile collector. For example, $\Phi_{s}\left(i,j\right)=1$ denotes that node *j* is the *i*th node visited by the walk. Then each element of $\Phi_{s}$ can be expressed as follows

(8) $$\Phi_{s}\left(i,j\right)=\left\{\begin{array}{cc} 1, & \;j\in V_{i}\\ 0, & \;otherwise. \end{array}\right.$$

where $V_{i}$ is the set of the vertices which is the *i*th visited node by the mobile collector.

### 4.3. Spatial-Temporal Sparsity Representation Bases Design

Due to spatial-temporal correlation of sensory data, we consider a two-dimensional sparsity representation basis to sparsify a sensing field. For time-series sensory data from one sensor node, we adopt DCT transform basis $\Psi_{t}$ to compress these data in temporal domain. For spatial correlation of sensory data, we use a kernel-based method to sparsify a sensing field as in [41]. A two-dimensional Gaussian kernel function is adopted to construct a transform basis in spatial domain as follows

(9) $$K\left(\mu_{i},\mu_{j}\right)=e^{-\frac{∥\mu_{i}-\mu_{j}{∥}^{2}}{2\omega^{2}}},$$

where $\mu_{i}\in R^{2}$ represents the coordinates of node *i* following an i.i.d. sample with uniform distribution on ${\left[0,1\right]}^{2}$. $∥\mu_{i}-\mu_{j}∥=d_{ij}$ represents the distance between node *i* and node *j*. We assume these distances are known. As a result, the corresponding kernel matrix $K_{n}$ can be expressed as follows

(10) $$K_{n}=\left[\begin{array}{cccc} e^{-\frac{d_{11}^{2}}{2\omega^{2}}} & e^{-\frac{d_{12}^{2}}{2\omega^{2}}} & \cdots & e^{-\frac{d_{1n}^{2}}{2\omega^{2}}}\\ e^{-\frac{d_{21}^{2}}{2\omega^{2}}} & e^{-\frac{d_{22}^{2}}{2\omega^{2}}} & \cdots & e^{-\frac{d_{2n}^{2}}{2\omega^{2}}}\\ \vdots & \vdots & \vdots & \vdots \\ e^{-\frac{d_{n1}^{2}}{2\omega^{2}}} & e^{-\frac{d_{n2}^{2}}{2\omega^{2}}} & \cdots & e^{-\frac{d_{nn}^{2}}{2\omega^{2}}} \end{array}\right].$$

We adopt a centered version of the kernel matrix where data is centered in the feature space. Each entry ${\tilde{K}}_{ij}$ of the centered kernel matrix ${\tilde{K}}_{n}$ is given as [47]

(11) $${\tilde{K}}_{ij}=K_{ij}-\frac{1}{n}\sum_{l=1}^{n}K_{lj}-\frac{1}{n}\sum_{l=1}^{n}K_{il}+\frac{1}{n^{2}}\sum_{l=1}^{n}\sum_{m=1}^{n}K_{lm},$$

where $K_{ij}$ is the entry of the uncentered kernel matrix $K_{n}$. We then diagonalize the kernel matrix ${\tilde{K}}_{n}$ as ${\tilde{K}}_{n}=\Psi \Lambda \Psi^{-1}$, where $\Psi_{s}$ is an orthonormal eigenvector basis, Λ is the diagonal matrix whose diagonal elements are the corresponding eigenvalues of ${\tilde{K}}_{n}$. In this paper, we use $\Psi_{s}$ as a representation basis to sparsify sensory data in spatial domain.

### 4.4. Reconstruction Algorithm of Spatial-Temporal Data

Let $x_{i}=\left(x_{1,i},x_{2,i},\ldots ,x_{m,i}\right)$ denote the measurement vector taken by node *i* and $x\in R^{m\times n}$ represent the matrix of all the measurements sampled by *n* nodes. Hence, x can be represented as a 2-D signal. In this work, we employ the framework of KCS for spatial-temporal sensory data [48].

As aforementioned, we use $\Psi_{s}$ and $\Psi_{t}$ as the sparsifying bases for spatial and temporal domain data, respectively. According to the theory of KCS, a single sparsifying basis for an entire multidimensional signal can be obtained by the Kronecker products of sparsifying bases for each dimensional signal [48]. Then, a single sparsifying basis for x can be obtained as $\bar{\Psi }=\Psi_{s}⊗\Psi_{t}$, which is defined as

(12) $$\bar{\Psi }:=\left[\begin{array}{ccc} \Psi_{t}\left(1,1\right)\Psi_{s} & \ldots & \Psi_{t}\left(1,m\right)\Psi_{s}\\ \vdots & \ddots & \vdots \\ \Psi_{t}\left(m,1\right)\Psi_{s} & \ldots & \Psi_{t}\left(m,m\right)\Psi_{s} \end{array}\right].$$

Hence, x can be represented as $x^{\prime }=\bar{\Psi }\theta$, where $x^{\prime }$ is a vector-reshaped representation of x and θ is the vector of transform coefficients of $x^{\prime }$.

Similarly, we can also use Kronecker products to develop measurement matrices. As discussed above, at each sensor node the matrix $\Phi_{t}$ is used to obtain its individual compressive measurements and the matrix $\Phi_{s}$ is used to obtain compressive measurements of all the sensor nodes, which is a boolean random matrix. Hence, the joint measurement matrix can be expressed as $\bar{\Phi }=\Phi_{s}⊗\Phi_{t}$ as follows

(13) $$\bar{\Phi }:=\left[\begin{array}{ccccc} \Phi_{t} & 0 & 0 & \ldots & 0\\ 0 & 0 & \Phi_{t} & \ldots & 0\\ \vdots & \vdots & \vdots & \ddots & \vdots \\ 0 & 0 & 0 & \ldots & \Phi_{t} \end{array}\right],$$

where 0 represents a matrix with all entries being 0.

Suppose that the compressive measurements obtained at the sink are denoted as $Y={\left(y_{1}^{T}y_{2}^{T}\ldots y_{m_{s}}^{T}\right)}^{T}$ where $m_{s}$ is the number of the nodes visited by the mobile collector. The data obtained by the sensor nodes is denoted as $X={\left(x_{1}^{T}x_{2}^{T}\ldots x_{n}^{T}\right)}^{T}$. Then, recovering the signal X from compressive measurements Y can be conducted by solving the following $\ell_{1}$-minimization problem:

(14) $$\min{∥\theta ∥}_{\ell_{1}}s.t.\;Y=\bar{\Phi }\bar{\Psi }\theta ,\;X=\bar{\Psi }\theta .$$

## 5. Algorithm and Performance Analysis

In this section, we first prove that the random CS matrix $A=\bar{\Phi }\bar{\Psi }$ constructed from the proposed spatial-temporal data gathering scheme with the MHDA algorithm and the spatial-temporal representation basis $\bar{\Psi }$, follows the RIP. Then, we analyze the performance of the proposed scheme in terms of the actual number of steps that the mobile collector needs to take during a data gathering tour.

### 5.1. Algorithm Analysis

We now prove the following lemma stating that the random CS matrix $A=\bar{\Phi }\bar{\Psi }$ follows the RIP with the following bounds of restricted isometry constants. The restricted isometry constant is defined as follows:

**Definition** **1.** *The K-restricted isometry constant δ for the matrix $A=\bar{\Phi }\bar{\Psi }$ is the smallest nonnegative number such that, for all $\theta \in R^{N}$ with ${\left||\theta |\right|}_{0}=K$*

(15) $${\left(1-\delta \right)||\theta ||}_{2}^{2}\leq {\left||A\theta |\right|}_{2}^{2}\leq {\left(1+\delta \right)||\theta ||}_{2}^{2}$$

**Lemma** **1.** *Let ${\bar{\Phi }}_{s}=\Phi_{s}\Psi_{s}$, ${\bar{\Phi }}_{t}=\Phi_{t}\Psi_{t}$ and $A=\bar{\Phi }\bar{\Psi }$, where $\bar{\Phi }=\Phi_{s}⊗\Phi_{t}$, $\bar{\Psi }=\Psi_{s}⊗\Psi_{t}$. If $\Phi_{s}$, $\Phi_{t}$ are defined as in Section 4.2 and $\Psi_{s}$, $\Psi_{t}$ are defined as in Section 4.3, then ${\bar{\Phi }}_{s}$ and ${\bar{\Phi }}_{t}$ satisfy the RIP with high probability, respectively, and*

(16) $$\max\left(\delta_{s},\delta_{t}\right)\leq \delta \left(A\right)\leq \left(1+\delta_{s}\right)\left(1+\delta_{t}\right)-1,$$

*where $\delta_{s}$ and $\delta_{t}$ are the restricted isometry constants of ${\bar{\Phi }}_{s}$ and ${\bar{\Phi }}_{t}$, respectively.*

**Proof.**  We first prove that both ${\bar{\Phi }}_{s}$ and ${\bar{\Phi }}_{t}$ follow the RIP with high probability. As discussed above, we adopt a random Gaussian or Bernoulli random matrix as the random measurement matrix $\Phi_{t}$ and the DCT transform basis as the orthonormal sparsity basis $\Psi_{t}$. Obviously, the random matrix ${\bar{\Phi }}_{t}=\Phi_{t}\Psi_{t}$ follows the RIP with high probability. We now are ready to prove that ${\bar{\Phi }}_{s}=\Phi_{s}\Psi_{s}$ also follows the RIP with high probability. The proof can be done following the similar reasoning as in [41]. Compared with [41], a different random walk algorithm is employed in this paper, which results in a distinct node sampling probability.

As proved in [41], the entries of $\Psi_{s}$ follow a sub-Gaussian distribution, i.e., $\Psi_{s}\left(j,i\right)∼Sub\left(c^{2}\right)$, where c>0 is a constant. Then, for any $x\in R^{N}$, $<\Psi_{s}\left(j,:\right),x>∼Sub(c^{2}{∥x∥}_{2}^{2})$ [49] Lemma 4.2. Let $Y=(Y_{1},Y_{2},\ldots ,Y_{m_{s}})$ and $E\left({\left(<\Psi_{s}\left(j,:\right),x>\right)}^{2}\right)=\sigma^{2}$, where $Y_{j}=<\Psi_{s}\left(j,:\right),x>$ for $j=1,\ldots ,m_{s}$. Then we have ${E(∥Y∥}_{2}^{2})=m_{s}\sigma^{2}$ [49] Theorem 4.2. As discussed above, the probability that each row is selected from ${\bar{\Phi }}_{s}$ is equal to the probability that the corresponding node is visited by the random walk. As proved in [45], the MHDA algorithm is theoretically guaranteed to construct a random walk algorithm to achieve a uniform stationary distribution, improving efficiency over the standard MHRW algorithm. According to the theorem in [49] Theorem 4.2, for any α∈(0,1) and $\beta \in [c^{2}/\sigma_{2},\beta_{\max}]$, there exists a constant $C^{*}\geq 4$ depending only on $\beta_{\max}$ and the ratio $\sigma^{2}/c^{2}$ such that

(17) $${\mathrm{Pr}(∥Y∥}_{2}^{2}\leq \alpha m_{s}\sigma^{2})\leq e^{-m_{s}{\left(1-\alpha \right)}^{2}/C^{*}}$$

and

(18) $${\mathrm{Pr}(∥Y∥}_{2}^{2}\geq \beta m_{s}\sigma^{2})\leq e^{-m_{s}{\left(\beta -1\right)}^{2}/C^{*}}.$$

By normalization, $\Phi_{s}^{\prime }=\sqrt{\frac{n}{m_{s}}}{\left({\bar{\Phi }}_{s1},{\bar{\Phi }}_{s2},\ldots ,{\bar{\Phi }}_{s_{m}}\right)}^{T}$. Furthermore, we have $E\left(\Phi_{s}^{\prime }{\left(j,i\right)}^{2}\right)={\left(\sqrt{\frac{n}{m_{s}}}\right)}^{2}E\left(\Phi_{s}^{\prime }{\left(j,i\right)}^{2}\right)=\frac{1}{m_{s}}$ and $E\left(\Phi_{s}^{\prime }\left(j,i\right)\right)=\sqrt{\frac{n}{m_{s}}}E\left(\Phi_{s}\left(j,i\right)\right)=0$. Then, we obtain

(19) $$\begin{array}{c} E(∥Y{∥}_{2}^{2})\\ =E(\sum_{j=1}^{m}{\left(\Phi_{s}^{\prime }\left(j,:\right)x\right)}^{2})\\ =\sum_{j=1}^{m}E{\left(\sum_{i=1}^{n}\left(\Phi_{s}^{\prime }\left(j,i\right)x_{i}\right)\right)}^{2}\\ =\sum_{j=1}^{m}(E\left(\Phi_{s}^{\prime }{\left(j,i\right)}^{2}\right){∥x∥}_{2}^{2}+2\sum_{i=1}^{n}\sum_{k\neq i}E\left(\Phi_{s}^{\prime }\left(j,i\right)\right)E\left(\Phi_{s}^{\prime }\left(j,i\right)\right)x_{i}x_{k})\\ ={∥x∥}_{2}^{2}. \end{array}$$

By setting α=1−δ, Equation (17) can be expressed as

(20) $$\begin{array}{cc} {\mathrm{Pr}(∥Y∥}_{2}^{2}\leq {\left(1-\delta \right)E(∥Y∥}_{2}^{2})) & \leq e^{-m_{s}\delta^{2}/C^{*}}\\ \mathrm{Pr}(∥{\bar{\Phi }}_{s}x{∥}_{2}^{2}\leq \left(1-\delta \right)∥x{∥}_{2}^{2}) & \leq e^{-m_{s}\delta^{2}/C^{*}}. \end{array}$$

Similarly, by setting β=1+δ, Equation (18) becomes

(21) $$\begin{array}{cc} \mathrm{Pr}(∥{\bar{\Phi }}_{s}x{∥}_{2}^{2}\geq \left(1+\delta \right)∥x{∥}_{2}^{2}) & \leq e^{-m_{s}\delta^{2}/C^{*}}. \end{array}$$

For a k-sparse signal x, there exist (n,k) possible signal sets x. Then we have $\left(n,k\right)\leq {\left(en/k\right)}^{k}$ by Sterling’s approximation. Therefore, we have the probability exceeding $1-2{\left(en/k\right)}^{k}\cdot e^{-\frac{m\delta^{2}}{C*}}=1-2e^{-\frac{m\delta^{2}}{C*}+k\log\left(n/k\right)+k}$ such that

(22) $$\begin{array}{c} \left(1-\delta_{s}\right){∥x∥}_{2}^{2}\leq {∥{\bar{\Phi }}_{s}x∥}_{2}^{2}\leq \left(1+\delta_{s}\right){∥x∥}_{2}^{2}. \end{array}$$

Therefore, we can choose $m_{s}=O\left(k\log\left(n/k\right)\right)$ such that ${\bar{\Phi }}_{s}$ satisfies the RIP with the probability approximating to 1.

From the above analysis, we have proved that ${\bar{\Phi }}_{s}$ and ${\bar{\Phi }}_{t}$ follow the RIP with high probability, respectively. According the property of Kronecker product, we have

(23) $$\begin{array}{cc} A & =\bar{\Phi }\bar{\Psi }\\ & =\left(\Phi_{s}⊗\Phi_{t}\right)\left(\Psi_{s}⊗\Psi_{t}\right)\\ & =\left(\Phi_{s}\Psi_{s}\right)⊗\left(\Phi_{t}\Psi_{t}\right)\\ & ={\bar{\Phi }}_{s}⊗{\bar{\Phi }}_{t}. \end{array}$$

Let $\delta_{s}$ and $\delta_{t}$ are the restricted isometry constants of ${\bar{\Phi }}_{s}$ and ${\bar{\Phi }}_{t}$, respectively. In [48], it has been proven that the RIP constant of the Kronecker product matrix $\delta ({\bar{\Phi }}_{s}⊗{\bar{\Phi }}_{t})$ satisfies the following inequality

(24) $$\max\left(\delta_{s},\delta_{t}\right)\leq \delta \left({\bar{\Phi }}_{s}⊗{\bar{\Phi }}_{t}\right)\leq \left(1+\delta_{s}\right)\left(1+\delta_{t}\right)-1.$$

Then, we obtain

(25) $$\max\left(\delta_{s},\delta_{t}\right)\leq \delta \left(A\right)\leq \left(1+\delta_{s}\right)\left(1+\delta_{t}\right)-1,$$

which finishes the proof. ☐

### 5.2. Performance Analysis

We are now ready to calculate the actual number of steps $m_{l}$ that the mobile collector needs to take to visit $m_{s}$ distinct sensor nodes. Since the mobile collector may visit a sensor node for several times including self loop steps the actual number of steps $m_{l}$ should be larger than $m_{s}$. The advantage of the MHDA algorithm is that it can avoid backtracking to the previously visited nodes to reduce the number of steps that it spends at the same nodes.

**Lemma** **2.** *The expected number of steps that the mobile collector needs to take to visit $m_{s}$ distinct sensor nodes over the graph G is*

(26) $$E\left(m_{l}\right)\leq m_{s}/\left(\frac{d_{\min}}{d_{\max}}+\frac{1}{d_{\max}}\cdot {\left(\frac{d_{\min}}{d_{\max}}\right)}^{3}\right)$$

*where $d_{\min}$ and $d_{\max}$ are the minimum and maximum degree of the graph G, respectively.*

**Proof.**  Consider a random walk with a length of $m_{l}$ using the MHDA algorithm and let $U_{1}$, $U_{2}$,*…*, $U_{m_{s}}$ be the distinct nodes that the mobile collector may visit during the random walk. Suppose that for a random walk and a sequence of nodes $v_{1},\;v_{2},\ldots ,\;v_{i}$, $U_{1}=v_{1},\;U_{2}=v_{2},\ldots ,\;U_{i}=v_{i}$. Let $X_{i}$ denote the number of steps that the mobile collector needs to spend at node $v_{i}$ until it visits the next distinct node. Hence, $X_{i}$ is 1 plus the number of steps that the mobile collector stays at the self loop. Note that $X_{i}$ is a random variable which only depends on $U_{i}$ and not on the previously visited node. Now consider any step at node $v_{i}$. Then, the probability of $X_{i}=1$ excluding the number of steps at the self loop is $d_{v_{i}}p_{ij}$, where $d_{v_{i}}$ is the degree of $v_{i}$ and $p_{ij}$ is the transition probability of the random walk. Hence, $E\left(X_{i}|U_{1}=v_{1},\;U_{2}=v_{2},\ldots ,\;U_{i}=v_{i},\ldots \right)=E\left(X_{i}|U_{i}=v_{i}\right)=\frac{1}{d_{v_{i}}p_{ij}}$. Furthermore, $\sum_{i=1}^{m_{s}}X_{i}=m_{l}$. Note that for every sequence of node $v_{1}$, $v_{2}$,*…*, the random variables $X_{1}$, $X_{2}$,*…* are independent given that $U_{1}=v_{1}$, $U_{2}=v_{2}$, *…* According to Wald’s identity,

(27) $$\begin{array}{c} E(\sum_{i=1}^{m_{s}}X_{i}|U_{1}=v_{1},U_{2}=v_{2},\ldots )\\ =E\left(m_{s}|U_{1}=v_{1},U_{2}=v_{2},\ldots \right)E\left(X_{i}|U_{1}=v_{1},U_{2}=v_{2},\ldots \right)\\ =\frac{m_{s}}{d_{v_{i}}p_{ij}}. \end{array}$$

Note that $m_{s}$ is a fixed number given before the random walk runs and $m_{l}$ is a random variable. So $E(m_{l})$ is the actual number of steps that we want to calculate. Since $\sum_{i=1}^{m_{s}}X_{i}=m_{l}$, we obtain:

(28) $$E\left(m_{l}\right)=\frac{m_{s}}{d_{v_{i}}p_{ij}}.$$

Since

(29) $$\begin{array}{cc} d_{v_{i}}p_{ij} & =d_{v_{i}}(\min\left\{\frac{1}{d_{v_{j}}},\frac{1}{d_{v_{i}}}\right\}+\min\left\{\frac{1}{d_{v_{j}}},\frac{1}{d_{v_{i}}}\right\}\cdot \frac{1}{d_{v_{j}}-1}\\ & \cdot \min\{1,\min\left\{\frac{1}{d_{v_{j}}^{2}},\frac{1}{d_{v_{k}}^{2}}\right\}/\min\left\{\frac{1}{d_{v_{j}}^{2}},\frac{1}{d_{v_{i}}^{2}}\right\})\\ & \geq \frac{d_{\min}}{d_{\max}}+\frac{d_{\min}}{d_{\max}}\cdot \frac{1}{d_{\max}}\cdot \frac{d_{\min}^{2}}{d_{\max}^{2}}, \end{array}$$

then we have

(30) $$E\left(m_{l}\right)\leq m_{s}/\left(\frac{d_{\min}}{d_{\max}}+\frac{1}{d_{\max}}\cdot {\left(\frac{d_{\min}}{d_{\max}}\right)}^{3}\right).$$

Note that the probability $p_{ij}$ is the transition probability of the random walk involving the two scenarios as described in the Algorithm 1. ☐

### 5.3. Discussion

In the following, we discuss the performance improvement of the proposed scheme over the other existing schemes in terms of the number of transmissions that the sensor nodes require to deliver their measurements to the mobile collector. Firstly, we take the raw data transmission without compression as a baseline scheme for comparison, where the measurements of *n* sensor nodes with each having *m* measurements are collected by a mobile collector by using a standard random walk algorithm until all of nodes have been visited. Obviously, it requires at least total mn transmissions in one round of data gathering. Additionally, it also requires O(mnlogn) time slots to visit all of nodes in a random geometric network, which has been proven in [50]. In the meanwhile, compared with the schemes using multiple random walks as proposed in [21,26,27], our scheme only needs to take a random walk, which consumes $E=O(k_{t}k_{s}\log\left(m/k_{t}\right)\log\left(n/k_{s}\right))$ transmissions and spends $T=O(k_{t}k_{s}\log\left(m/k_{t}\right)\log\left(n/k_{s}\right))$ time slots, whereas it takes $m_{r}=O\left(k_{s}\log\left(n/k_{s}\right)\right)$ random walks with a length of $T_{r}=O\left(n/k_{s}\right)$ steps [21], resulting in total $E=O(k_{t}n\log\left(m/k_{t}\right)\log\left(n/k_{s}\right))$ number of transmissions. Therefore, the proposed scheme has significant improvements on energy consumption and data gathering delay over the existing CS-based approaches.

However, we note that it is not efficient to use only one single mobile collector for data gathering, since it incurs high delay due to the low moving velocity of the mobile collector. To further reduce the collection time, multiple mobile collectors can be employed so that each mobile collector may visit fewer sensor nodes to collect their measurements. On the other hand, we also note that the proposed scheme might incur high cost due to the deployment of mobile collector. Such a mobile collector requires high power to allow it to move around a sensing field. However, it in turn helps to save energy consumption at each sensor node since it is possible for a node to use a relatively small power of its transceiver for data transmission when a mobile collector comes by.

## 6. Numerical Simulations

In this section, we evaluate the performance of the proposed scheme for spatial-temporal data gathering in WSNs through simulations. The proposed algorithm is implemented by MATLAB. A cvx package is used to solve $\ell_{1}$ programming for CS decoding algorithm [51].

### 6.1. Spatial and Temporal Correlation Characteristics of the Dataset

**Figure 3:**
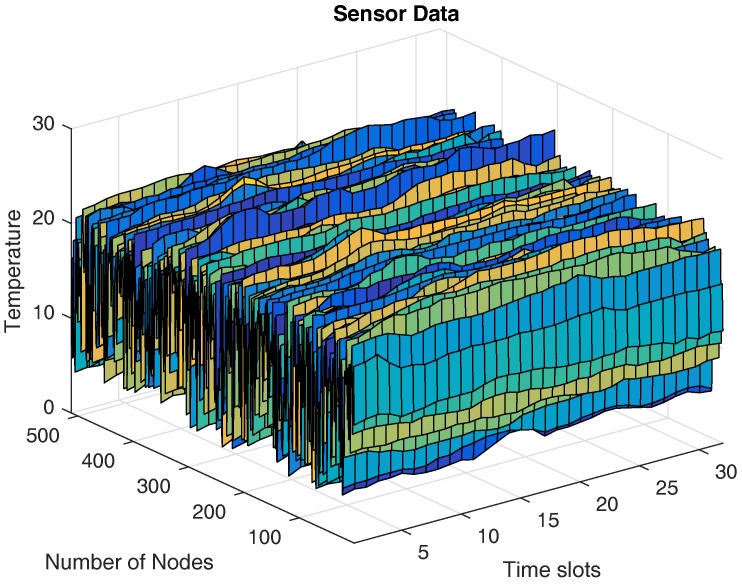
The spatial-temporal temperature data collected by 512 sensor nodes.

**Figure 4:**
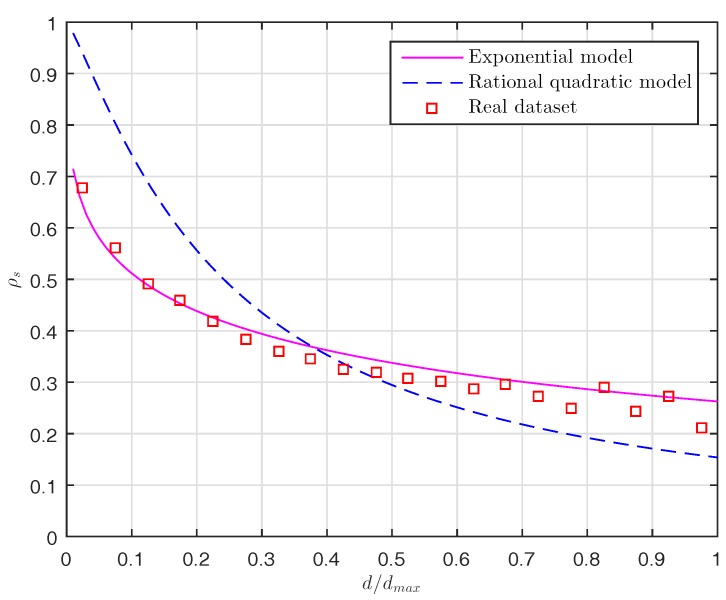
Spatial correlation for the real dataset and the two correlation fitting models (PE and RQ).

In this section, we first introduce the dataset adopted in the simulation and then analyze the correlation characteristics. The real dataset we use is obtained from a remote sensing system to measure sea surface temperature [52]. We assume that a wireless sensor network is composed of 512 sensor nodes which are randomly and uniformly deployed in a sensing field to monitor the sea environment. Such a deployment will result in an irregular network topology. Figure 3 presents spatial-temporal temperature data collected by the sensor nodes. To analyze the correlation characteristics of the real dataset, we compute the spatial correlation and the temporal correlation of the dataset following the approach proposed by Zordan et al. in [53]. In order to calculate the spatial correlation, we randomly select 3000 pairs of points from the total number of pairs. For each pair of points, we calculate its distance *d* and the corresponding spatial correlation function $\rho_{S}$ using Equation (2) in [53]. Similarly to the procedure adopted in [53], we divide the maximum distance $d_{max}$ of the all pairs of points into 20 intervals. Then we calculate the average spatial correlation coefficients for all the pairs of points whose distance falls within the same interval. We also calculate the spatial correlation vs. the distance using the Power Exponential (PE) model and the Rational Quadratic (RQ) model [53]. In this simulation, we select the parameters ξ=0.38 and ν=0.3 for the PE model and ξ=1.8 and ν=1.2 for the RQ model (corresponding to Equations (3) and (4) in [53], respectively). In Figure 4, we plot the spatial correlation $\rho_{S}$ with the normalized distance $d/d_{max}\in \left[0,1\right]$ for the real dataset. As shown in Figure 4, we can see that the spatial correlation of the real dataset used in this paper nicely fits the PE model. On the other hand, we also calculate the temporal correlation coefficients of the dataset using Equation (6) in [53]. We find that the average temporal correlation coefficient of the dataset is 0.9984, which shows the strong temporal correlation.

### 6.2. Performance of Spatial-Temporal Sparsity Representation Basis

**Figure 5:**
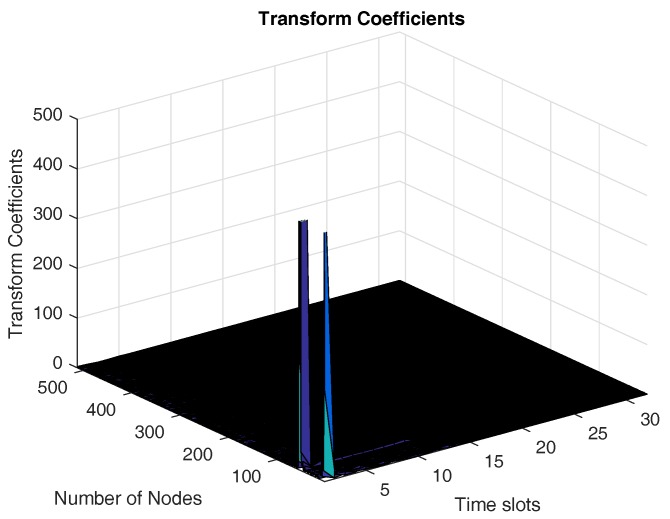
The transform coefficients employing GKB-DCT as a 2D transform basis.

In this section, we evaluate the performance of the proposed scheme using spatial-temporal sparsity basis and show the performance improvement over the other conventional bases. We use Gaussian kernel basis (GKB) and DCT as the spatial and temporal transform bases (GKB-DCT), respectively. The kernel parameter ω is set to 1 for GKB. The relative error is used to evaluate the reconstruction quality, which is defined as $\varepsilon =∥\hat{x}{-x∥}_{2}^{2}/{∥x∥}_{2}^{2}$, where x and $\hat{x}$ are the original sensory data and the reconstructed data, respectively. Figure 5 shows 2D transform coefficients by using GKB-DCT. From Figure 5, it can be seen that there are only few transform coefficients whose absolute values are much larger than the remaining coefficients. This explains that GKB-DCT can be well used to sparsify sensory data for an irregular topology. We also compare the reconstruction performance with the other schemes using spatial sparsity bases such as Laplacian eigenvector basis (LEB), discrete wavelet transform (DWT) and discrete cosine transform (DCT) and temporal sparsity basis DCT (termed as LEB-DCT, DWT-DCT, DCT-DCT), respectively. The eigenvector of the Laplacian matrix of a graph G(V,E) has been commonly adopted to sparsify data for an irregular topology [21,54]. The Laplacian Matrix of a graph is usually used to characterize the topology of a sensor network, which is defined as follows [55]:

$$L=\left\{\begin{array}{c} -1\;\mathrm{if}\;(i,j)\in E\\ d_{i,i}\;\mathrm{if}\;i=j\\ 0\;\mathrm{otherwise}, \end{array}\right.$$

where $d_{i,i}$ is the degree of node *i*.

**Figure 6:**
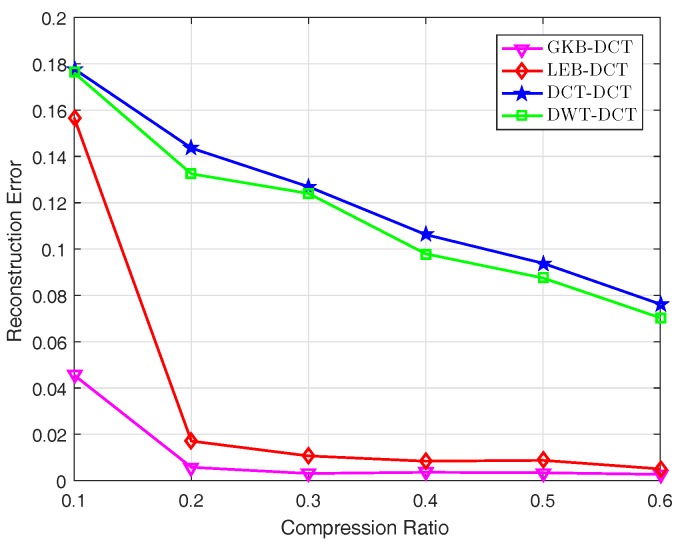
The reconstruction error under different transform bases.

From Figure 6, it is noticed that the proposed scheme using GKB-DCT outperforms the other schemes using LEB-DCT, DWT-DCT, DCT-DCT. This indicates that GKB-DCT is a more efficient spatial-temporal transform basis to sparsify data than other bases in an irregular deployment. Figure 6 also shows that the conventional bases (e.g., DCT and DWT) perform poorly. This is because such bases are more appropriate for a regular deployment.

### 6.3. Performance of Spatial-Temporal Data Gathering with Mobile Collector

**Figure 7:**
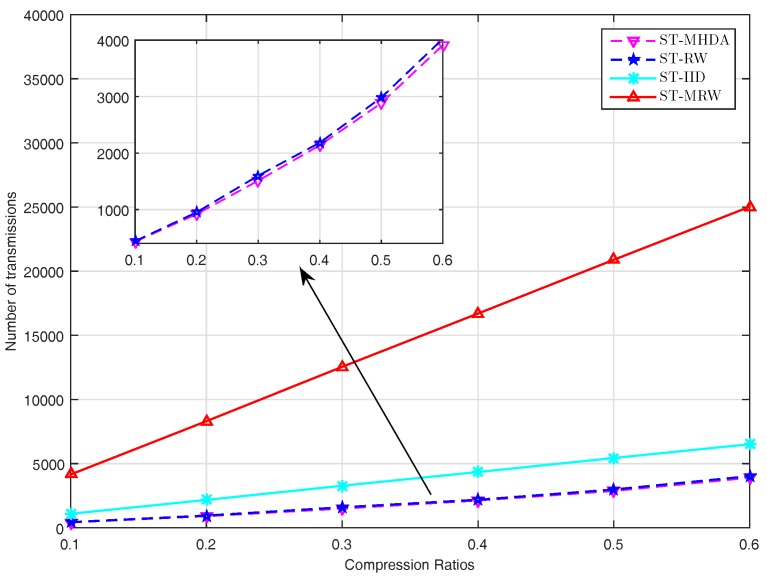
The number of transmissions required for different CS-based schemes.

In this section, we evaluate the performance of the proposed spatial-temporal data gathering scheme using the MHDA algorithm. We choose $r\left(n\right)=\sqrt{\frac{8\log n}{n}}$ as the detection range of the mobile collector since it has been proven that a random geometric graph has optimal partial cover time with high probability if $r\left(n\right)\geq \sqrt{\frac{8c\log n}{n}}$ where c≥1 [50]. We first investigate the performance for data gathering without considering temporal data under the varying compression ratios. We compare our scheme with the other schemes using simple random walk (RW) algorithm, IID algorithm and dense random projections (DRPs). In the RW scheme, the mobile collector randomly and uniformly selects one node from its neighbors for the next visit. In the IID scheme, we assume that the fusion center randomly picks up some of the nodes to send their data. In the DRPs scheme, we employ a Bernoulli random matrix instead of using a mobility model to generate a random measurement matrix as the baseline scheme for performance comparison. We use the Gaussian kernel basis as the sparsity representation basis for all the schemes. Figure 7 plots the reconstruction error for the schemes above. The reconstruction error is computed over 20 trials for each scheme. From Figure 7, we observe that the proposed MHDA algorithm outperforms the RW algorithm when compression ratios vary from 0.1 to 0.3. This is because the node sampling distribution induced by the MHDA algorithm is more uniform than the one induced by the RW algorithm. The IID algorithm outperforms the MHDA algorithm due to the same reason. However, we also notice that when compression ratios vary from 0.4 to 0.6, the MHDA algorithm can achieve the similar reconstruction quality compared to the IID and DRPs schemes.

We next compare the performance in terms of communication cost of sensor nodes for the four schemes above. The communication cost is evaluated in terms of the number of transmissions required for the sensor nodes to send their data to the mobile collector or the fusion center. Figure 7 plots the number of transmissions required for different schemes. We can see that the proposed scheme outperforms all of the other schemes. For instance, our scheme can provide about on average 4%, 49% and 87% transmission cost reduction compared to ST-SRW, ST-IID, ST-MRW. Compared to ST-SRW, the performance gain of our scheme benefits from the fact that the MHDA algorithm makes the mobile collector avoid backtracking the previously visited nodes.

**Figure 8:**
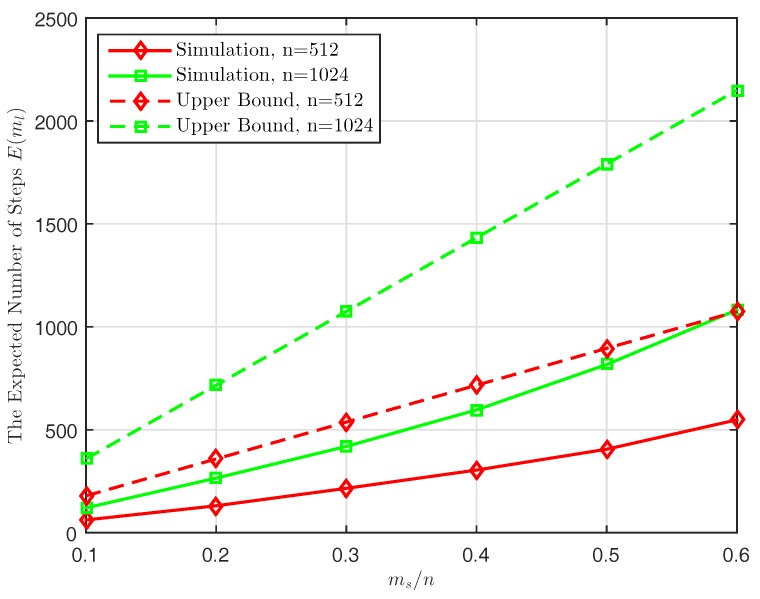
The expected number of steps $m_{l}$ taken by mobile collector to visit $m_{s}$ distinct sensor nodes.

We also conduct simulations to validate the analytical result derived in Lemma 2. As discussed before, since the mobile collector may backtrack to some previously visited nodes, it needs to take additional steps that it spends at the same nodes. In this simulation, we calculate the actual number of steps $m_{l}$ taken by the mobile collector to visit $m_{s}$ distinct sensor nodes under the various sizes of network *n*. Figure 8 plots the expected number of steps $E(m_{l})$ and the upper bound of the analytical result (i.e., the right term in Equation (30)) with the parameter $m_{s}/n$ for n=512 and n=1024, respectively. It can be seen that the actual number of steps $E(m_{l})$ is upper bounded by the analytical result, which is approximately smaller than $m_{s}d_{\max}/d_{\min}$. In [56], it has been proven that the maximum and minimum degree of a connected random geometric graph *G* are in the same order, i.e, $d_{\max}/d_{\min}=c$ where *c* is a constant. This indicates that the actual number of steps $E(m_{l})$ approximately scales linearly with the number of distinct nodes $m_{s}$ and has the same order with $m_{s}$ (i.e., $E\left(m_{l}\right)=O\left(m_{s}\right)$).

**Figure 9:**
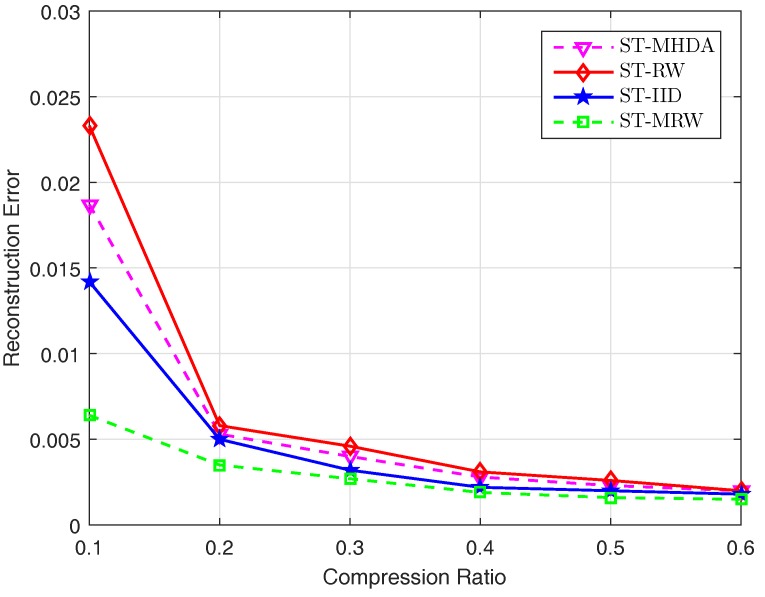
The reconstruction error for spatial-temporal data collection with different CS-based schemes.

We then compare the performance with various spatial-temporal data gathering schemes including ST-SRW, ST-IID, ST-MRW. ST-SRW and ST-IID extend the aforementioned schemes SRW and IID to the spatial-temporal case. In the ST-MRW scheme, we extend the multiple random walks based algorithm adopted in [21] to the spatial-temporal data collection scheme where an LEB-DCT transform basis is adopted. Figure 9 shows the reconstruction performance for the four schemes above. We observe the similar results as obtained in the only spatial data collection scheme above.

On the other hand, instead of using multi-hops as in ST-IID or multiple random walks as in ST-MRW, the proposed scheme performs only a random walk for data collection, which is able to significantly reduce the communication cost of sensor nodes.

**Figure 10:**
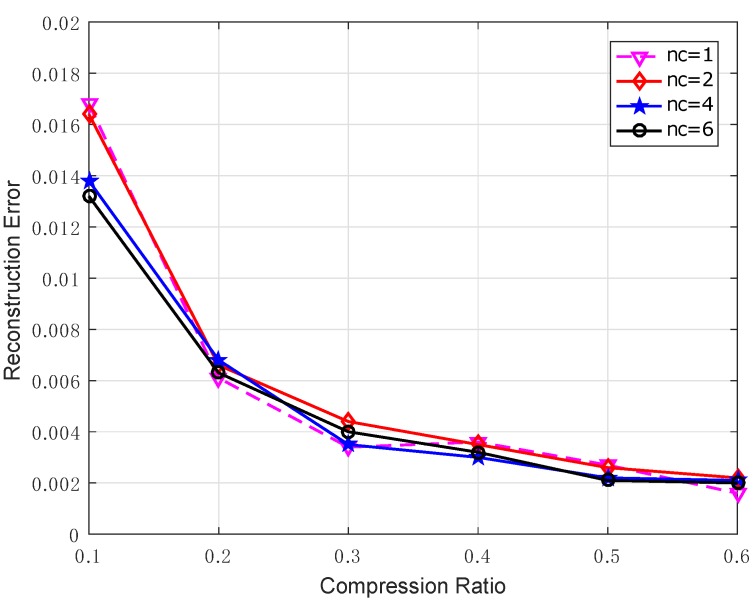
The reconstruction error for the proposed scheme with multiple mobile collectors.

On the other hand, it is worth noting that it might be not efficient by using only one single mobile collector for data gathering since it incurs high delay. In this simulation, we investigate the performance of employing multiple mobile collectors for data collection. Obviously, we can make use of multiple mobile collectors to increase the speed of data collection since each one may visit fewer sensor nodes to collect the measurements. Figure 10 plots the reconstruction error when the number of mobile collectors is nc=1,2,4,6, respectively. It is clear that the similar reconstruction quality can be achieved when the total number of measurements collected by all the mobile collectors in each scheme is the same. Therefore, our scheme can also be easily extended to the case using multiple collectors to reduce the data gathering delay although only one collector is utilized in this work.

### 6.4. Performance of the Proposed Scheme with Packet Loss

**Figure 11:**
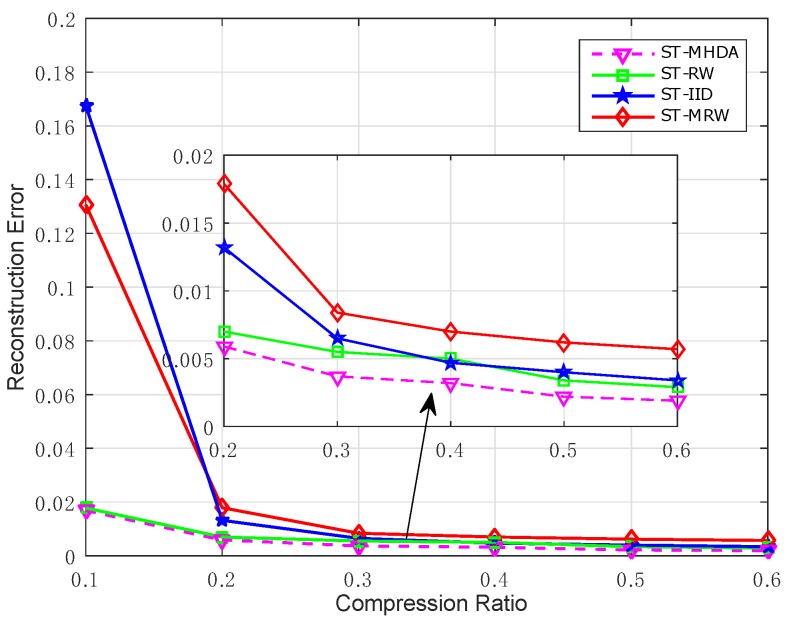
A comparison of the reconstruction error for different CS-based schemes under packet loss.

We further evaluate the performance of the proposed scheme against unreliable wireless channel environments. In this simulation, we assume that the temporal data at each sensor node in one round of data gathering is compressed by CS and encapsulated into a packet. We also assume that a packet at a sensor node is transmitted to the mobile collector with a packet loss rate *P*. we compare the performance of various spatial-temporal data gathering schemes under a 20% packet loss rate. As shown in Figure 11, the performance of the proposed scheme is superior to the other schemes compared to ST-RW, ST-IID, ST-MRW. This is due to the fact that a projection in our scheme is generated from only one sensor node instead of a linear combination of the measurements from multiple nodes as in ST-MRW. The result also demonstrates the mobile data gathering schemes outperform the multi-hop transmission strategies as adopted in ST-IID because any packet loss during the transmission to the fusion center through multi-hops will increase the loss probability of a packet.

## 7. Conclusions and Future Work

In this paper, we studied an energy-efficient data gathering scheme using compressive sensing for spatial-temporal sensory data in mobile wireless sensor networks. We proposed a novel spatial-temporal data gathering scheme using the Metropolis-Hastings random walk algorithm with delayed acceptance, which allows a mobile collector to harvest compressive measurements by sequentially visiting a small subset of nodes along a random routing path. We proved that the equivalent sensing matrix constructed from the proposed scheme for spatial-temporal dimensional compressible signal satisfies the RIP. In particular, we showed that from the mobile collector needs to visit $m_{s}=O\left(k_{s}\log\left(n/k_{s}\right)\right)$ randomly selected nodes and collect $m_{t}=O\left(k_{t}\log\left(m/k_{t}\right)\right)$ compressive measurements from each node so as to reconstruct a sensing field assuming that the field has $k_{s}$ and $k_{t}$ spatial and temporal dimensional sparsity, respectively. We presented extensive simulation results to demonstrate that the proposed scheme is able to not only significantly reduce communication cost but also improve reconstruction accuracy compared to some existing CS-based schemes. We also showed that the proposed scheme be also resilient to unreliable wireless environment under various packet losses. However, we shall note that it requires accurate location information of sensor nodes to construct a sparsity representation basis for signal recovery due to the use of Gaussian kernel basis. On the other hand, the mobile collector also requires these location information for the navigation in the sensing field. When these location information is not available, how to develop a more efficient sparsity representation basis and a more practical random walk algorithm may be more important for a wireless sensor network. As a result, we intend to leave it for future study.

## References

[1] Wu M., Tan L., Xiong N. (2016). Data prediction, compression, and recovery in clustered wireless sensor networks for environmental monitoring applications. Inf. Sci..

[2] Huang K., Zhang Q., Zhou C., Xiong N., Qin Y. (2017). An efficient intrusion detection spproach for visual sensor networks based on traffic pattern learning. IEEE Trans. Syst. Man Cybern. Syst..

[3] Xiong N., Vasilakos A.V., Yang L.T., Song L., Pan Y., Kannan R., Li Y. (2009). Comparative analysis of quality of service and memory usage for adaptive failure detectors in healthcare systems. IEEE J. Sel. Areas Commun..

[4] Wu P., Xiao F., Sha C., Huang H., Wang R., Xiong N. (2017). Node scheduling strategies for achieving full-view area coverage in camera sensor networks. Sensors.

[5] Yuen K., Liang B., Li B. (2008). A distributed framework for correlated data gathering in sensor networks. IEEE Trans. Veh. Technol..

[6] Hua G., Chen C.W. (2008). Correlated data gathering in wireless sensor networks based on distributed source coding. Int. J. Sens. Netw..

[7] Shen G., Ortega A. Optimized distributed 2d transforms for irregularly sampled sensor network grids using wavelet lifting. Proceedings of the 2008 IEEE International Conference on Acoustics, Speech, and Signal Processing (ICASSP).

[8] Xu X., Li X.-Y., Wan P.-J., Tang S. (2012). Efficient scheduling for periodic aggregation queries in multihop sensor networks. IEEE/ACM Trans. Netw..

[9] Donoho D. (2006). Compressed sensing. IEEE Trans. Inf. Theory.

[10] Luo C., Wu F., Sun J., Chen C.W. Compressive data gathering for large-scale wireless sensor networks. Proceedings of the 15th Annual International Conference on Mobile Computing and Networking (ACM Mobicom).

[11] Luo C., Wu F., Sun J., Chen C.W. (2010). Efficient measurement generation and pervasive sparsity for compressive data gathering. IEEE Trans. Wirel. Commun..

[12] Xiang L., Luo J., Rosenberg C. (2013). Compressed data aggregation: Energy-efficient and high-fidelity data collection. IEEE/ACM Trans. Netw..

[13] Zheng H., Xiao S., Wang X., Tian X. Energy and latency analysis for in-network computation with compressive sensing in wireless sensor networks. Proceedings of the IEEE INFOCOM (Mini-Conference).

[14] Zheng H., Xiao S., Wang X., Tian X., Guizani M. (2013). Capacity and delay analysis for data gathering with compressive sensing in wireless sensor networks. IEEE Trans. Wirel. Commun..

[15] Xie R., Jia X. (2014). Transmission efficient clustering method for wireless sensor networks using compressive sensing. IEEE Trans. Parallel Distrib. Syst..

[16] Zhao C., Zhang W., Yang Y., Yao S. (2015). Treelet-based clustered compressive data aggregation for wireless sensor networks. IEEE Trans. Veh. Technol..

[17] Nguyen M., Teague K.A., Rahnavard N. Inter-cluster multi-hop routing in wireless sensor networks employing compressive sensing. Proceedings of the Military Communications Conference (MILCOM).

[18] Quer G., Masiero R., Munaretto D., Rossi M., Widmer J., Zorzi M. On the interplay between routing and signal representation for compressive sensing in wireless sensor networks. Proceedings of the Information Theory and Applications Workshop (ITA).

[19] Lee S., Pattem S., Sathiamoorthy M., Krishnamachari B., Ortega A. Spatially-localized compressed sensing and routing in multi-hop sensor networks. Proceedings of the Third International Conference on Geosensor Networks.

[20] Sartipi M., Fletcher R. Energy-efficient data acquisition in wireless sensor networks using compressed sensing. Proceedings of the IEEE Data Compression Conference (DCC).

[21] Zheng H., Yang F., Gan X., Tian X., Wang X., Xiao S. (2015). Data gathering with compressive sensing in wireless sensor networks: A random walk based approach. IEEE Trans. Parallel Distrib. Syst..

[22] Liu X., Zhu Y., Kong L., Liu C., Gu Y., Vasilakos A.V., Wu M. (2015). CDC: Compressive data collection for wireless sensor networks. IEEE Trans. Parallel Distrib. Syst..

[23] Wu X., Xiong Y., Yang P., Wan S., Huang W. Compressive sensing meets unreliable link: Sparsest random scheduling for compressive data gathering in lossy WSNs. Proceedings of the 15th ACM International Symposium on Mobile Ad Hoc Networking and Computing.

[24] Zhang H., Zhu Y., Tan J. Adaptive sampling and sensing approach with mobile sensor networks. Proceedings of the IEEE International Conference on Cyber Technology in Automation, Control and Intelligent Systems.

[25] Wang Q., Lv C., Shen Y., Chen J. Compressed sensing and mobile agent based sparse data collection in wireless sensor networks. Proceedings of the IEEE International Instrumentation and Measurement Technology Conference (I2MTC).

[26] Nguyen M.T., Teague K.A. (2015). Random walk based data collection in mobile sensor networks utilizing compressed sensing. Int. J. Complex Syst. Comput. Sens. Control.

[27] Nguyen M.T., Teague K.A. Random sampling in collaborative and distributed mobile sensor networks utilizing compressive sensing for scalar field mapping. Proceedings of the 10th System of Systems Engineering Conference (SoSE).

[28] Mahmudimanesh M., Khelil A., Suri N. Balanced spatio-temporal compressive sensing for multi-hop wireless sensor networks. Proceedings of the IEEE 9th International Conference on Mobile Ad-Hoc and Sensor Systems.

[29] Quer G., Masiero R., Pillonetto G., Rossi M., Zorzi M. (2012). Sensing compression and recovery for WSNs: Sparse signal modeling and monitoring framework. IEEE Trans. Wirel. Commun..

[30] Xu X., Ansari R., Khokhar A. Spatio-temporal hierarchical data aggregation using compressive sensing (ST-HDACS). Proceedings of the 2015 International Conference on Distributed Computing in Sensor Systems.

[31] Wang Y., Yang Z., Zhang J., Li F., Wen H., Shen Y. (2016). CS^2^-collector: A new approach for data collection in wireless sensor networks based on two-dimensional compressive sensing. Sensors.

[32] Li X., Tao X., Chen Z. (2017). Spatio-temporal compressive sensing based data gathering in wireless sensor networks. IEEE Wirel. Commun. Lett..

[33] Wang S., Yi H., Wu L., Zhou F., Xiong N. (2017). Mining probabilistic representative gathering patterns for mobile sensor data. J. Internet Technol..

[34] Cheng H., Su Z., Xiong N., Xiao Y. (2016). Energy-efficient nodes scheduling algorithms for wireless sensor networks using Markov Random Field model. Inf. Sci..

[35] Chang C., Chen G., Yu G., Wang T.L., Wang T.C. (2015). TCWTP: Time-constrained weighted targets patrolling mechanism in wireless mobile sensor networks. IEEE Trans. Syst. Man Cybern. Syst..

[36] La H.M., Sheng W., Chen J. (2015). Cooperative and active sensing in mobile sensor networks for scalar field mapping. IEEE Trans. Syst. Man Cybern. Syst..

[37] Lee C., Kwak J., Kong L., Eun D. (2016). Towards distributed optimal movement strategy for data gathering in wireless sensor networks. IEEE Trans. Parallel Distrib. Syst..

[38] Fan Q., Zeitouni K., Xiong N., Wu Q., Camtepe S., Tian Y. (2016). Nash equilibrium-based semantic cache in mobile sensor grid database systems. IEEE Trans. Syst. Man Cybern. Syst..

[39] Nguyen M.T., Teague K.A. Compressive wireless mobile sensing for data collection in sensor networks. Proceedings of the IEEE International Conference on Advanced Technologies for Communications (ATC).

[40] Rana R., Yang M., Wark T., Chou C.T., Hu W. (2015). Simpletrack: Adaptive trajectory compression with deterministic projection matrix for mobile sensor networks. IEEE Sens. J..

[41] Zheng H., Guo W., Xiong N. (2017). A Kernel-based compressive sensing approach for mobile data gathering in wireless sensor network systems. IEEE Trans. Syst. Man Cybern. Syst..

[42] Candès E.J., Tao T. (2006). Near-optimal signal recovery from random projections: Universal encoding strategies?. IEEE Trans. Inf. Theory.

[43] Candès E., Romberg J., Tao T. (2006). Robust uncertainty principles: Exact signal reconstruction from highly incomplete frequency information. IEEE Trans. Inf. Theory.

[44] Shah R.C., Roy S., Jain S., Brunette W. (2003). Data MULEs: Modeling and analysis of a three-tier architecture for sparse sensor networks. Ad Hoc Netw..

[45] Lee C.H., Xu X., Eun D.Y. Beyond random walk and metropolis-hastings samplers: Why you should not backtrack for unbiased graph sampling. Proceedings of the SIGMETRICS Performance.

[46] Zhao J., Xi W., He Y., Liu Y., Li X.Y., Mo L., Yang Z. (2013). Localization of wireless sensor networks in the wild: Pursuit of ranging quality. IEEE/ACM Trans. Netw..

[47] Liang Z. (2014). Eigen-Analysis of Kernel Operators for Nonlinear Dimension Reduction. Ph.D. Thesis.

[48] Duarte M.F., Baraniuk R.G. (2012). Kronecker compressive sensing. IEEE Trans. Image Process..

[49] Davenport M.A. (2010). Random Observations on Random Observations: Sparse Signal Acquisition and Processing. Ph.D. Thesis.

[50] Avin C., Ercal G. (2007). On the cover time and mixing time of random geometric graphs. Theor. Comput. Sci..

[51] Grant M., Boyd S. CVX: Matlab Software for Disciplined Convex Programming. http://cvxr.com/cvx/.

[52] National Center for Atmospheric Research Staff Last Modified 6 May 2013. The Climate Data Guide: SST (AMSR-E): Sea Surface Temperature from Remote Sensing Systems. https://climatedataguide.ucar.edu/guidance/sst-amsr-e-sea-surface-temperature-remote-sensing-systems.

[53] Zordan D., Quer G., Zorzi M., Rossi M. Modeling and generation of space-time correlated signals for sensor network fields. Proceedings of the IEEE Global Telecommunications Conference (GLOBECOM).

[54] Dimakis A.G., Kar S., Moura J.M.F., Rabbat M.G., Scaglione A. (2010). Gossip algorithms for distributed signal processing. Proc. IEEE.

[55] Chung F. (1997). Spectral Graph Theory. CBMS Regional Conference Series in Mathematics.

[56] Xue F., Kumar P.R. (2004). The number of neighbors needed for connectivity of wireless networks. Wirel. Netw..

